# Antibiotic-associated changes in *Akkermansia muciniphila* alter its effects on host metabolic health

**DOI:** 10.1186/s40168-024-02023-4

**Published:** 2025-02-07

**Authors:** Yumin Han, Teh Min Teng, Juwon Han, Heenam Stanley Kim

**Affiliations:** Division of Biosystems & Biomedical Sciences, College of Health Sciences, 145 Anam-Ro, Seongbuk-Gu, Seoul, 02841 Korea

**Keywords:** *Akkermansia muciniphila*, Gut microbiome, Antibiotic resistance, Persistence, TEM-type β-lactamase, The de novo purine biosynthesis pathway, *pur* gene, Obesity

## Abstract

**Background:**

Altered gut microbiota has emerged as a major contributing factor to the etiology of chronic conditions in humans. Antibiotic exposure, historically dating back to the mass production of penicillin in the early 1940s, has been proposed as a primary contributor to the cumulative alteration of microbiota over generations. However, the mechanistic link between the antibiotics-altered microbiota and chronic conditions remains unclear.

**Results:**

In this study, we discovered that variants of the key beneficial gut microbe, *Akkermansia muciniphila*, were selected upon exposure to penicillin. These variants had mutations in the promoter of a TEM-type β-lactamase gene or *pur* genes encoding the de novo purine biosynthesis pathway, and they exhibited compromised abilities to mitigate host obesity in a murine model. Notably, variants of *A. muciniphila* are prevalent in the human microbiome worldwide.

**Conclusions:**

These findings highlight a previously unknown mechanism through which antibiotics influence host health by affecting the beneficial capacities of the key gut microbes. Furthermore, the global prevalence of *A. muciniphila* variants raises the possibility that these variants contribute to global epidemics of chronic conditions, warranting further investigations in human populations.

Video Abstract

**Supplementary Information:**

The online version contains supplementary material available at 10.1186/s40168-024-02023-4.

## Background

In recent decades, there has been a noticeable increase in various chronic conditions worldwide, a major cause of which has been suggested to be altered gut microbiota in modern human populations [1–5]. Industrialization and urbanization have been proposed to have profoundly and cumulatively contributed to the alteration of the microbiota over generations [1, 2, 6, 7]. This notion has been supported by studies comparing human populations at different levels of modernization [6, 8, 9], and has been demonstrated experimentally in mice [10, 11]. Elements of industrialization and urbanization include cleaner water, smaller families, increased Caesarean births, lower rates of breastfeeding, dietary changes (particularly reduced fiber consumption), and antibiotic uses [7, 10, 12]. Among these, frequent and excessive exposure to antibiotics, dating back to the mass production of penicillin in the early 1940s, has been suggested as one of the most significant contributors to microbiota alteration [1, 3, 4, 13]. Consistent with this notion, antibiotics have been demonstrated to disrupt microbiota [13–15]. Moreover, such disruptions have been shown to lead to improper responses of colonic regulatory T cells, compromised immune tolerance, and increased susceptibility to various disorders, including asthma, allergy, autoimmunity, obesity, and vulnerability to opportunistic infections [13–17]. Widely used taxonomic-level profiling using sequencing technology provides an overall picture of the altered community structure of the gut microbiota. However, assessing additional changes at the individual cellular level, including the emergence of resistance mutations and the induction of stringent responses, is necessary to comprehensively understand the consequences of antibiotics-altered gut microbiota [18]. Such a cellular-level impact may be particularly pronounced when a key beneficial microbe, such as *Akkermansia muciniphila*, is involved. *A. muciniphila* is a prevalent gut bacterium that resides in the mucus layer of the human colon [19, 20]. It has the potential to mitigate various chronic conditions, including obesity, type 2 and type 1 diabetes mellitus, hepatic steatosis, intestinal inflammation, and certain cancers [21–24]. Consequently, the potential therapeutic applications of this bacterium have sparked extensive research worldwide [25–27].

In this study, we investigated the effect of penicillin, an initially mass-produced antibiotic that was potentially overused extensively during the early stages of the antibiotic era [28], on *A. muciniphila*. We discovered that variants of *A. muciniphila* were selected for survival upon exposure to penicillin, and that the variants exhibited diminished host-beneficial capacities when assessed in a murine model. These findings provide a novel perspective on how antibiotics influence host health by affecting the key members of the gut microbiota.

## Methods

### Bacterial strains and cultures

*Akkermansia muciniphila* strain BAA-835 (MucT) [19] was acquired from ATCC (American Type Culture Collection). An *A. muciniphila* isolate was obtained from the feces of a 24-year-old healthy female residing in Seoul, Korea, and was designated strain HM1. *A. muciniphila* strains were cultured in the BHIM medium, which is BHI (brain heart infusion) (Bacto Laboratories, Mt. Pritchard, NSW, Australia) broth supplemented with 0.4% (w/v) mucin from porcine stomach type II (Sigma-Aldrich, St. Louis, MO, USA). *A. muciniphila* cultures were incubated at 37 ℃, in an anaerobic chamber (Coy Laboratory Products, Grass Lake, MI, USA) that maintained anaerobic conditions (N_2_ 85%, CO_2_ 10%, H_2_ 5%). Fully grown cultures were mixed with a 50% volume of sterile, deoxygenated glycerol and stored in a −80 ℃ freezer until needed.

### Antibiotic selection of *A. muciniphila* variants

The entire process was performed under anaerobic conditions in an anaerobic chamber. *A. muciniphila* strain HM1 was grown on BHIM agar plates at 37 ℃ for 4 days. A single colony was used to inoculate 3 ml of BHIM broth, and the culture was incubated for 40 h at 37 °C. The bacterial cells were centrifuged, washed with fresh BHIM broth, and adjusted to approximately ${10}^{7}$ CFU/ml. A total of 100 μl of the bacterial suspension was spread on BHIM agar plates containing penicillin G (6–12 μg/ml), set at 3–4 times the MIC against *A. muciniphila* strain HM1. The plates were then incubated for 7 days at 37 °C until colonies were formed. These colonies were streaked onto fresh selective agar plates to confirm the acquired antibiotic resistance.

### Identification of mutations conferring reduced antibiotic susceptibility

The genome of strain HM1 (wild type) was sequenced using the Sequel I platform (Pacific Biosciences, Menlo Park, CA, USA) and supplemented with error correction through the Illumina NovaSeq 6000 platform (Illumina, San Diego, CA, USA) at DNA Link (DNA Link Inc., Seoul, Korea). Genomic DNA was extracted using the MagAttract High Molecular Weight (HMW) DNA kit (Qiagen, Germantown, CA, USA) following the manufacturer’s instructions. The DNA was then fragmented into 10-kb pieces using a Covaris G-tube (Covaris Inc., Woburn, MA, USA), again according to the manufacturer’s protocol. A SMRTbell library was prepared from the sheared DNA and sequenced on the Sequel I platform. To improve the PacBio results, the genomic DNA from strain HM1 was re-sequenced using the Illumina NovaSeq 6000. A DNA library was prepared with the TruSeq Nano DNA library Prep Kit (Illumina, San Diego, CA, USA) following manufacturer’s instructions and sequenced with paired-end 150 bp reads. Raw sequencing data were demultiplexed using Lima v2.9.0 (https://lima.how/). High-quality reads were assembled de novo with Flye v2.9-b1774 (https://github.com/fenderglass/Flye), followed by genome polishing for error correction using Pilon v1.23 (https://github.com/broadinstitute/pilon/wiki). Assembly completeness was assessed with BUSCO v5.4.4 (https://gitlab.com/ezlab/busco). Genome annotation was carried out using the NCBI Prokaryotic Genome Annotation Pipeline (https://github.com/ncbi/pgap). The final genome sequence for strain HM1 was deposited in GenBank under accession number CP16946.

To identify mutations in *A. muciniphila* variants that confer reduced antibiotic susceptibility, the genomes of these variants were sequenced using the HiSeq-X platform (Illumina, San Diego, USA) [29] to an average depth of approximately 360 to 420X. Data trimming, read mapping to the reference genome, and duplicate read removal were performed using Trimmomatic (v0.38) [30], BWA (v0.7.17) [31], and Sambamba (v0.6.7) [32], respectively. Single nucleotide polymorphism (SNP) analysis was conducted with SAMtools (v1.9) [33] and BCFtools (v1.6) [34]. SNPs and short indel candidates with Phred scores above 30 (base call accuracy of 99.9%) were identified using information from the mapped reads.

### Etest analyses with *A. muciniphila* strains

The antibiotic susceptibility of *A. muciniphila* strains (WT, p*-βL, PurF*, and PurM*) was measured with the Epsilometer test (Etest®) gradient technology (Biomerieux, Marcy-l’Etoile, France). The Etest strips of amoxicillin (AC), ampicillin (AM), benzylpenicillin (penicillin G, PG), and ceftazidime (CZ) were used in the concentration range of 0.016 ~ 256 μg/ml (AC, AM, and TZ) and 0.002 ~ 32 μg/ml (PG). Single colonies from the plates were suspended in 2 ml BHI broth until the turbidity reached 4.0 McFarland standard. Using sterile cotton swabs, cell suspensions were spread on the agar plates of BHI supplemented with mucin (0.4%). After drying the surfaces of the plates for 15 to 20 min, Etest strips were placed on the plates. The plates were incubated for 2 ~ 6 days (or until a sufficient bacterial growth was observed) at 37 °C, and the MIC values were scored.

### Growth analysis

A single colony of each *A. muciniphila* strain was used to inoculate 3 ml BHIM broth, and the culture was incubated at 37 ℃ in an anaerobic chamber until it reached the stationary phase (e.g., OD_600_ = ~ 0.8 for the WT). The bacterial culture was then diluted 1:100 in 40 ml fresh BHIM broth and was incubated at 37 ℃. The optical density at 600 nm (OD_600_) was measured periodically using a Nanodrop One cuvette (Thermo Fisher Scientific, Waltham, MA, USA). To evaluate the effects of N-acetyl-D-glucosamine and hypoxanthine on mutant strains of *A. muciniphila*, 10 mM of N-acetyl-D-glucosamine (Sigma-Aldrich, Inc. St. Louis, MO, USA) or 4 mM of hypoxanthine (Sigma-Aldrich, Inc. St. Louis, MO, USA) was added to BHIM broth before conducting the assays. The experiment was repeated three times, and the average values, along with the standard error, were calculated and used to plot the data on a graph.

### Quantitative real-time PCR

*A. muciniphila* strains HM1 and p*-βL were cultured in 10 ml BHIM broth at 37 ℃ in an anaerobic chamber until they reached the stationary phase (OD_600_ = 0.7 ~ 1.2). Subsequently, the cultures were used to inoculate 80 ml fresh BHIM broth at a ratio of 1:50 and incubated at 37 ℃ until they reached OD_600_ of 0.4. Then, the cultures were divided, and each of the aliquots was supplemented with penicillin G (final concentration of 1 μg/ml) as a potential inducer for the TEM-type β-lactamase gene at a level lower than the MIC values for HM1 (2.7 μg/ml) or p*-βL (> 32 μg/ml). Cultures with or without the inducer were incubated at 37 ℃, and 1 ml samples were taken at 0 and 1 h and were immediately mixed with 2 ml RNA protect bacteria reagent (Qiagen Sciences Inc., Germantown, MD, USA). The mixtures were centrifuged at 5000 × g for 10 min, and the supernatants were discarded. Total RNA was extracted from each cell pellet using the RNeasy Mini Kit (Qiagen Sciences Inc., Germantown, MD, USA) according to the manufacturer’s instructions. The total RNA samples were converted into cDNA using M-MLV Reverse Transcriptase (Thermo Fisher Scientific, Waltham, MA, USA) following standard protocols. Quantitative real-time PCR (qRT-PCR) was performed to measure the expression level of the TEM-type β-lactamase gene of *A. muciniphila* strains HM1 and p*-βL using Rotor-Gene Q (Qiagen Sciences Inc., Germantown, MD, USA). Primer pairs used for the qRT-PCR of the TEM-type β-lactamase gene were 5′-GGAGCCCTGAATCTGGAACA-3′ and 5′-CACGTGTTGTTGTCGCTCTC-3′, and 5′-AGGACCTCGTTCTTGCCAAG-3′ and 5′-CAGGGTCTTGATCAGCACGT-3′ for *rpoB*. Fold changes in gene expression of the TEM-type β-lactamase gene, relative to the housekeeping gene *rpoB*, were determined using the comparative threshold cycle (*C*_*T*_) method [35].

For qPCR analysis of fecal samples collected from mice on the final day of bacterial administration, approximately 100 mg of feces was transferred into a 2.0-ml screw-cap tube containing 500 mg of 0.1 mm zirconia-silica beads (Biospec Products, Bartlesville, OK, USA). The tube was supplemented with 1 ml of 80% methanol and 5 µl of a 2-ethylbutyric acid stock solution. The mixture was homogenized using a Mini-Beadbeater-24 (Biospec Products) at 2400 rpm for 4 min, followed by a 4-min cooling period. This cycle was repeated three times. After homogenization, the samples were centrifuged at 14,000 rpm for 5 min at 4℃, and genomic DNA was extracted from the samples using the QIAamp PowerFecal Pro DNA kit (Qiagen, Hilden, Germany). Subsequent qRT-PCR analysis was conducted, as described above, to measure the relative abundance of *Akkermansia* DNA to total bacterial DNA. To detect *Akkermansia*, the primers AM1 (5′- CAGCACGTGAAGGTGGGGAC-3′) and AM2 (5′- CCTTGCGGTTGGCTTCAGAT-3′) were used [36]. The universal primer pairs Uni331F (5′- CAGCACGTGAAGGTGGGGAC-3′) and Uni797R (5′- CCTTGCGGTTGGCTTCAGAT-3′) were used to quantify the total microbiota [37].

### RNA-seq transcriptome analysis

Bacterial cultures of the WT strain and mutants were grown in BHIM medium at 37 ℃ until the OD_600_ reached 0.4. At this point, 1 ml of each culture was collected and mixed with 2 ml of RNAprotect Bacteria Reagent (Qiagen Sciences Inc., Germantown, MD, USA). The mixtures were centrifuged at 5000 × g for 10 min, and the supernatants were discarded. Total RNA was extracted from the cell pellets using the RNeasy Mini kit (Qiagen Sciences Inc., Germantown, MD, USA), following the manufacturer’s instructions. The RNA quality was assessed using the TapeStation4000 System (Agilent Technologies, Amstelveen, The Netherlands), and RNA quantification was performed with the ND-2000 Spectrophotometer (Thermo Inc., DE, USA). Bacterial RNA libraries were prepared with the CORALL RNA-Seq V2 Library Prep Kit (LEXOGEN, Inc., Austria), and rRNA was removed using the RIBO COP rRNA depletion kit (LEXOGEN, Inc., Austria). The rRNA-depleted RNA was used for cDNA synthesis and shearing according to the manufacturer’s instructions. Indexing was performed with Illumina indexes 1–12, and the enrichment step was conducted via PCR. The libraries were then checked using an Agilent 2100 bioanalyzer (DNA High Sensitivity Kit) to assess the mean fragment size. Quantification was done with the library quantification kit on a StepOne Real-Time PCR System (Life Technologies, Inc., USA). High-throughput sequencing was carried out as paired-end 100 bp reads on the NovaSeq 6000 platform (Illumina, Inc., USA) at e-biogen (Seoul, Korea).

Quality control of the raw sequencing data was performed using FastQC (https://www.bioinformatics.babraham.ac.uk/projects/fastqc/). Adapter and low-quality reads were removed with Fastp [38]. The trimmed reads were then mapped to the reference genome using STAR [39], and read quantification was carried out with Salmon [40]. Read counts were normalized using the TMM + CPM method in EdgeR [41]. Genes significantly altered in mutants compared to the WT strain were visualized using scatter plots, and gene ontology (GO) enrichment analysis was performed with Shiny GO (http://bioinformatics.sdstate.edu/go/).

### Mice and diets used to establish a diet-induced obesity mouse model

A total of 118 4-week-old male C57BL/6N mice were purchased from Koatech (Pyeongtaek, Korea). For the entire 12 weeks of the experiment, mice were housed in a facility with controlled temperature-humidity and a 12-h light–dark cycle. Water and diet were provided ad libitum. Mice were fed a normal diet (ND; Altromin International, # 1314; with 14% fat, 27% protein, and 59% carbohydrates; 3.34 kcal/g) or a high-fat diet (HFD; Research diets, # D12492; 60% fat, 20% protein, and 20% carbohydrates; 5.24 kcal/g). The fiber content was 6.46% in the high-fat diet (HFD) and 4.5% in the normal diet (ND), indicating that the difference in fiber intake was not substantial enough to have a significant impact. Body weight was measured once weekly.

### Oral gavage administration of bacterial cultures

After 7 weeks of growth (11 weeks-old mice), the mice were randomly divided into 7 groups (*n* ≧ 10 per group): normal diet only (referred to as ND only); high-fat diet only control (referred to as HFD only); HFD-fed MucT gavaged (referred to as *m*_MucT); HFD-fed WT gavaged (referred to as *m*_WT); HFD-fed p*-βL gavaged (referred to as *m*_p*-βL); HFD-fed PurF* gavaged (referred to as *m*_PurF*); and HFD-fed PurM* gavaged (referred to as *m*_PurM*). For the 5 weeks, mice were subjected to daily oral gavage using an 18-gauge rounded/bulb-tipped gavage needle to inject 200 µl of *A. muciniphila* culture grown in BHIM broth or just BHIM broth for mice in ND only and HFD only groups. The range of cells used for bacterial administration (approximately 2 × 10^8^ ~ 10^9^ cells per 200 μl) was carefully maintained as a standard. Each day, cultures across all strains were adjusted to achieve the most similar cell numbers possible within this range by concentrating or diluting the cultures. Although pasteurized cells can be as effective, or even more effective, than live cells [27, 42], we opted to use live cells for a more natural approach. Bacterial cultures for administration were prepared as follows. First, 20 ml of each strain was cultured anaerobically in BHIM broth at 37 ℃ until reaching the log phase (OD_600_ = 0.4). The cultures were then mixed with an equal volume of sterile, deoxygenated glycerol, aliquoted into 0.1 ml samples, and stored at −80 ℃. Before each bacterial administration, these master stocks (0.1 ml) were used to inoculate 10 ml of fresh BHIM broth, and the cultures were grown again to the log phase (OD_600_ = 0.4). The cell density was then adjusted to approximately 2 × 10^8^ ~ 10^9^ cells per 200 µl by centrifugation and resuspension in the medium for administration.

### Oral glucose tolerance test assays

After 5 weeks of daily gavage administration of *A. muciniphila* strains, mice underwent oral glucose tolerance test (OGTT) [43, 44]. The mice were subjected to an 8-h diet restriction period prior to the test. The baseline glucose concentration was measured before oral administration of glucose (2 g/kg body weight). Following glucose intake, blood was drawn from the tail vein, and the glucose concentration (mg/dl) was measured using an ACCU-CHEK GUIDE ME glucometer (Roche Diagnostics, Switzerland) at 15-min intervals over a period of 120 min. Measurements were performed in triplicate, and the area under the curve (AUC) [44] was calculated using the trapezoid rule.

### Sampling blood, liver, and adipose tissues

The mice were fasted overnight for 16 h and anesthetized with isoflurane before blood or adipose tissue sampling. Blood was collected from the abdominal caval veins using a 1-ml syringe and left for 0.5 ~ 1 h in a BD Microtainer Blood Collection tube (SKU: 365,967, Becton, Dickinson and Company, Franklin Lakes, NJ, USA) at room temperature. Blood serum was separated from the whole blood by centrifugation at 8000 × g, at 4 ℃ for 10 min. The serum was collected, separated into aliquots, and stored at −80 °C for later use. The epididymal, inguinal, retroperitoneal, perirenal, and mesenteric fat tissues were carefully dissected and weighed. The livers and kidneys were dissected and weighed. Adipose tissues were placed on plastic-coated paper with a 5 × 5-mm grid after washing in sterile 1 × PBS (phosphate buffered saline), and images were taken for documentation. After washing in sterile 1 × PBS, liver samples were fixed in 10% neutral-buffered formalin solution for histological analyses. Tissues were stored at −80 °C for later use.

### Analyses of serum LPS levels

The endotoxin lipopolysaccharide (LPS) level was measured using a Pierce Chromogenic Endotoxin Quantification Kit (Thermo Fisher Scientific, Waltham, MA, USA), following the manufacturer’s instructions. The serum was diluted (approximately 20- to 100-fold) in endotoxin-free water. All measurements were performed in duplicate. Marker concentrations were obtained by averaging the two measured concentrations and multiplying them by the dilution factor.

### Histological examination

Formalin-fixed liver samples were embedded in paraffin and sliced into 4-μm-thick sections. The slides were stained with Harris hematoxylin and eosin Y solution (H&E; Sigma-Aldrich, Burlington, MA, USA). Frozen liver tissue samples were embedded in an optimal cutting temperature (OCT) compound (Sakura Finetek USA, Inc., Torrance, CA, USA). The OCT-embedded samples were sectioned at 4 μm and stained with Oil Red O and hematoxylin (Sigma-Aldrich, Burlington, MA, USA). Stained sections were examined and imaged using an optical microscope (BX43F; Olympus Global, Tokyo, Japan).

### Statistical analysis

For all data, *p*-values were calculated using the Kruskal–Wallis test, followed by Dunn’s multiple comparison test. Statistical significance is indicated by asterisks (*): *p < 0.05, **p < 0.01, ***p < 0.001, and ****p < 0.0001.

### Screening the NCBI database for the DNA signatures for potential antibiotics-selected mutations

**Fig. 1:**
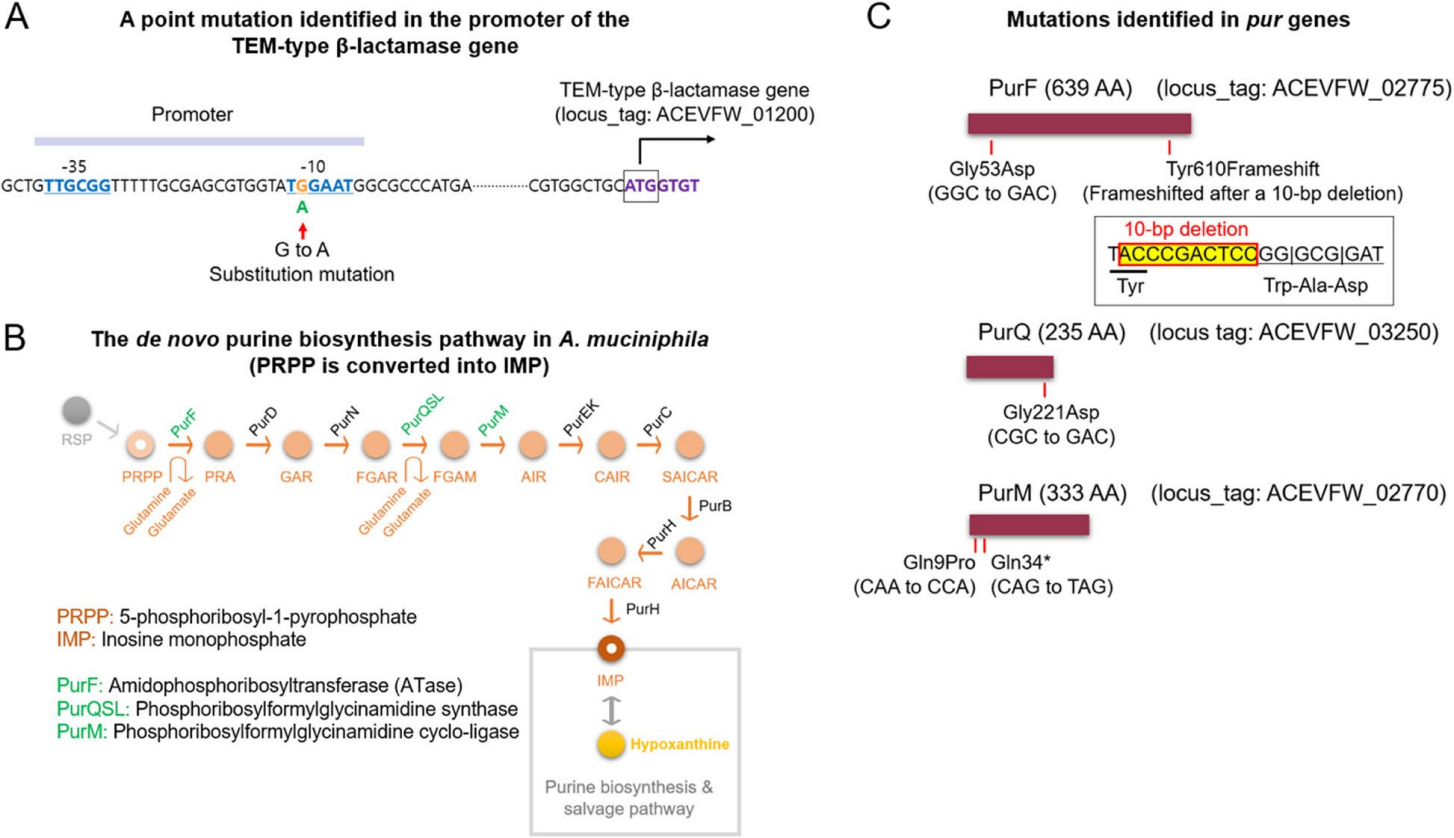
The identified mutations in the penicillin-selected variants of *Akkermansia muciniphila*. **A** A mutation identified in the promoter of a TEM-type β-lactamase gene (ACEVFW_01200). A single nucleotide substitution of G to A, which enhances the functionality of the −10 sequence, is shown. **B** The de novo purine biosynthesis pathway in *A. muciniphila* that was disrupted during antibiotic selection. The affected enzymes, PurF, PurQSL, and PurM, are denoted in green letters at their respective enzymatic steps. **C** Details of the mutations identified in *pur* genes. The two mutations in *purF* are a single-nucleotide substitution resulting in Gly53Asp and a ten-nucleotide deletion causing a frameshift, leading to consecutive amino acid substitutions starting at Tyr610. A single mutation in *purQ* is a single-nucleotide substitution leading to Gly221Asp, while two mutations in *purM* are single-nucleotide substitutions resulting in Gln9Pro or Gln34Nonsense, leading to premature termination of the enzyme

To explore the potential global distribution of the promoter mutation of the TEM-type β-lactamase gene identified in this study (Fig. 1A), we conducted BLASTN searches against the NCBI database (https://blast.ncbi.nlm.nih.gov/Blast.cgi) using a DNA sequence (200 bp) covering the promoter region and the 5ʹ-end portion of the TEM-type β-lactamase gene. Sequences from *A. muciniphila* strains carrying the A allele at the SNP position in the −10 sequence within the promoter were identified. Associated TEM-type β-lactamase homologs were retrieved from the NCBI. They were used to construct a phylogenetic tree initially by aligning the AA sequences using CLUSTALX 2.1 with the “Do Complete Alignment” option and default settings, then by uploading the final file to the ITOL website (https://itol.embl.de/) for tree construction and annotation. To identify strains with mutated Pur enzymes, segments of approximately 50 amino acid sequences from both the N-terminal and middle portions of PurF, PurQ, PurS, PurL, and PurM were used for BLASTP searches in the NCBI database. Proteins significantly shorter than their wild-type counterparts were retrieved, and their corresponding genes were inspected to identify frameshift or nonsense mutations.

## Results

### Variants of *A. muciniphila* were selected upon exposure to antibiotics

To investigate the impact of antibiotics on *A. muciniphila*, we exposed a strain of the bacterium isolated from a healthy individual (referred to as strain HM1, or simply the wild type (WT) in this study) to a lethal dose of penicillin G (6 ~ 12 μg/ml). The genomes of the WT strain and the variants that underwent in vitro selection were sequenced to identify genetic changes in the variants. Among the nine variants obtained, one had a mutation in the region upstream of a TEM-type β-lactamase gene (ACEVFW_01200), which appeared to be the promoter of the gene (Fig. 1A). This mutation involves a single-nucleotide substitution, in which G is replaced with A. This was within the presumed −10 sequence of the promoter, with a high potential to enhance promoter activity by increasing the AꞏT content of the −10 sequence [45] (Fig. 1A).

Additionally, in the remaining eight variants, we identified mutations in three *pur* genes, namely *purF*, *purQ*, and *purM* (Fig. 1B). These genes encode enzymes in the de novo purine biosynthesis pathway, a major pathway in nucleotide metabolism [46, 47] (Fig. 1B). *purF* encodes amidophosphoribosyltransferase (ATase), which catalyzes the initial step in the pathway converting 5-phosphoribosyl-1-pyrophosphate (PRPP) into inosine monophosphate (IMP) [46, 47] (Fig. 1B). *purQ*, along with *purS* and *purL*, encodes phosphoribosylformylglycinamidine synthase, whereas *purM* encodes phosphoribosylformylglycinamidine cycloligase. Eleven genes encoded the de novo purine biosynthesis pathway (Fig. 1B), similar to their homologs in *Escherichia coli* [46, 47]. The *pur* genes were distributed across the *A. muciniphila* chromosome and organized into multiple operons (Fig. S1). Two mutations were found in *purF*: a single-nucleotide substitution resulting in Gly53Asp and a ten-nucleotide deletion that caused a frameshift, resulting in the replacement of C-terminal 30 amino acids, starting at Tyr610 (Fig. 1C). Notably, this frameshift mutation occurred in two variants (Fig. 1C). The mutation found in *purQ* was a single-nucleotide substitution resulting in Gly221Asp (Fig. 1C). The two mutations identified in *purM* were single-nucleotide substitutions, resulting in Gln9Pro and premature termination at Gln34, representing a very early point in the enzyme (Fig. 1C). This premature termination occurred due to the conversion of the Gln-coding CAG into a nonsense codon T(U)AG (Fig. 1C). Notably, the Gln9Pro mutation in *purM* occurred in three variants (Fig. 1C). Unlike frameshift or nonsense mutations, the impact of single amino acid substitutions needs to be experimentally examined to determine their extent of effects. However, considering that the Gln9Pro mutation in *purM* occurred in three variants and that missense mutations also occurred in *purF* or *purQ* in other variants, it is likely that these missense mutations resulted in substantial disruptions in the respective enzymes, similar to frameshift or nonsense mutations, thereby impacting the purine biosynthesis pathway (Fig. 1C).

**Fig. 2:**
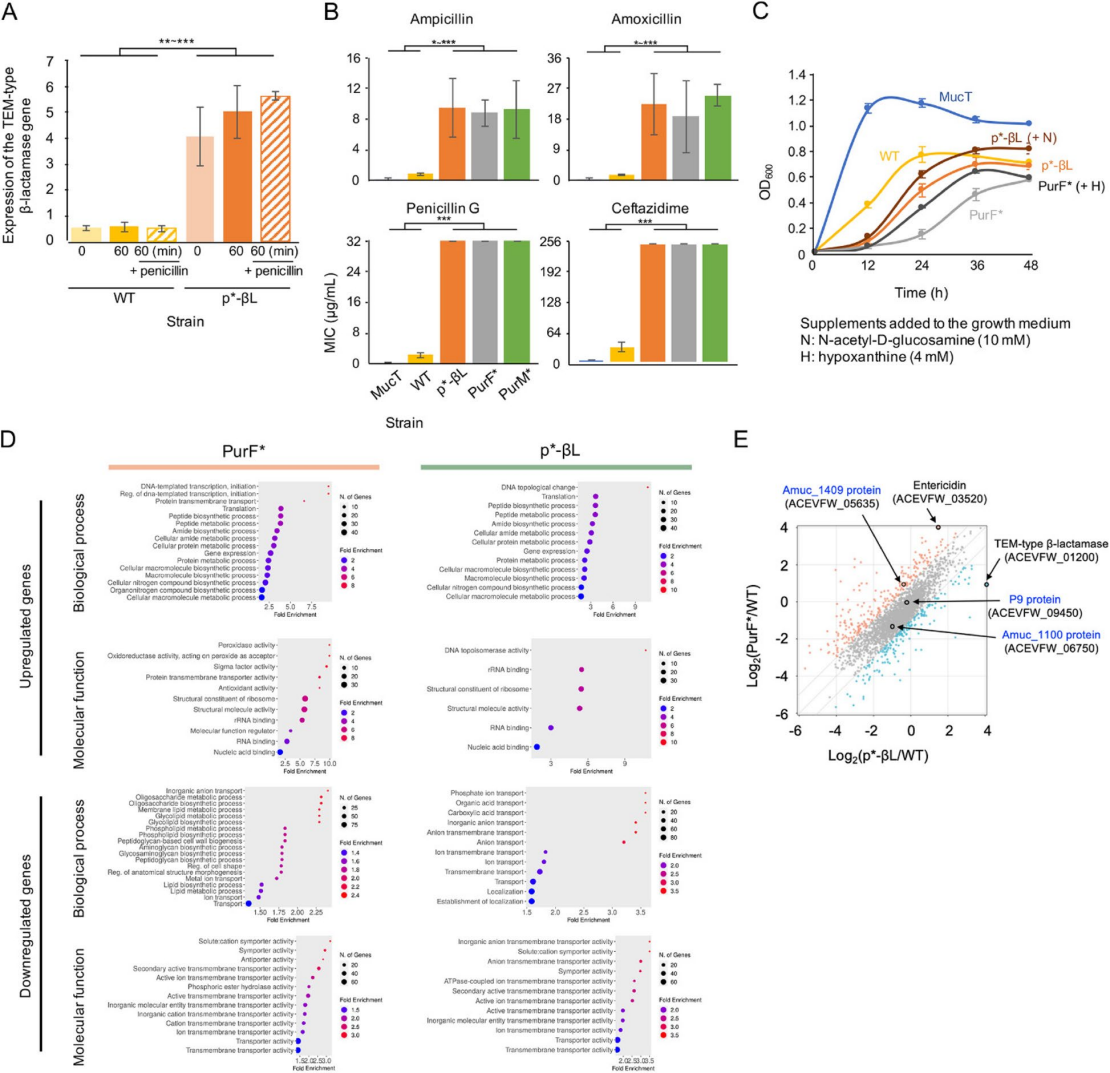
The antibiotics-selected *A. muciniphila* variants exhibited reduced susceptibility to antibiotics. **A** Quantitative PCR (qPCR) assays conducted with the WT and a strain featuring the promoter mutation, designated as p*-βL (mean ± S.D., *n* ≥ 3). Fold changes in gene expression of the TEM-type β-lactamase gene, relative to the housekeeping gene *rpoB*, are shown. ** *p* < 0.01, *** *p* < 0.001 (Kruskal–Wallis test). **B** Etest results of the variants. p*-βL and two strains carrying mutated *pur* genes, PurF* (with *purF* (Tyr610Frameshift)) and PurM* (with *purM* (Gln9Pro)), are compared to the WT (mean ± S.D., *n* ≥ 3). * *p* < 0.05, ** *p* < 0.01, *** *p* < 0.001 (Kruskal–Wallis test). **C** Growth curves of the antibiotics-selected variants compared to the WT (mean ± S.D., *n* ≥ 3). **D** Gene ontology (GO) enrichment analysis among the genes differentially expressed in PurF* and p*-βL compared to the WT. The data include genes that were upregulated or downregulated by at least twofold in each mutant strain. **E** Scatter plots of differentially expressed genes in PurF* and p*-βL compared to the WT. The data points for key genes that were affected or unaffected in the mutants are highlighted

To assess whether the predicted enhancement of promoter activity occurred due to the G to a single-nucleotide substitution in the putative −10 sequence, we conducted quantitative PCR (qPCR) assays with a variant strain carrying this mutation (designated as p*-βL) and the WT strain. The results demonstrated that the mutation significantly increased the basal-level expression of the TEM-type β-lactamase gene, as measured at two different time points (0 and 1 h) during incubation of the bacterial cultures within the log phase (Fig. 2A). Half of the cultures exposed to low levels of penicillin G (final concentration of 1 μg/ml) did not exhibit a significant induction in the gene regardless of the presence of the mutation (Fig. 2A). Consistent with the qPCR results, Etest results with strain p*-βL exhibited significantly elevated minimum inhibitory concentrations (MICs) for penicillin G, and additional β-lactam antibiotics, such as amoxicillin, ampicillin, and ceftazidime, compared to the WT (Fig. 2B). Additionally, two strains carrying a mutation in two *pur* genes, PurF* (carrying *purF* (Tyr610Frameshift)) and PurM* (carrying *purM* (Gln9Pro)), were assessed for their susceptibility to these antibiotics. The Etest results showed significantly reduced susceptibility of both variants to antibiotics compared to the WT (Fig. 2B). Similar to the WT, the type strain MucT (ATCC BAA-835) [19, 21] also exhibited high susceptibility to these antibiotics (Fig. 2B). The growth of the mutants was compared to that of the WT strain and MucT (Fig. 2C). Notably, MucT exhibited superior growth in BHIM broth compared to the WT strain (Fig. 2C). The growth of strain p*-βL was nearly identical to that of the WT strain, while PurF* exhibited significantly slower growth compared to the WT strain, suggesting a severely affected physiology of these purine mutant (Fig. 2C). The TEM-type β-lactamase gene is linked to additional downstream genes within the operon, one of which encodes acyl-ACP-UDP-N-acetylglucosamine O-acetyltransferase, an enzyme potentially involved in the utilization of N-acetyl-D-glucosamine, a core component of mucin [48] (Fig. S2). Supporting this notion, the supplementation of 10 mM N-acetyl-D-glucosamine to the growth medium significantly increased the rate and total amount of growth of the p*-βL strain, while no significant changes were observed in the WT or PurF* (data not shown). Similarly, supplementation with hypoxanthine, a key intermediate in purine biosynthesis and salvage pathway, which is interconvertible with inosine monophosphate (IMP) (Fig. 2B), stimulated the growth of PurF* (Fig. 2C). This supplementation did not significantly affect the WT or p*-βL (data not shown). To assess the effects of the mutations at the genomic level, we compared the transcriptomic profiles of the mutants p*-βL and PurF* with that of the WT (Fig. 2D). Both mutants exhibited a substantial number of upregulated genes compared to the WT, along with an even larger number of downregulated genes (Fig. 2D). PurF* demonstrated significant enrichment in various gene ontology (GO) terms, including transcription- and translation-related ones, among the upregulated genes, while p*-βL showed similar enrichment, though lacking transcription-related terms (Fig. 2D). Notably, PurF* exhibited enriched GO terms associated with activities against reactive oxygen species (ROS), suggesting that it was experiencing significant oxidative stress (Fig. 2D). This is consistent with previous reports showing that inefficiencies in ATP production, which can result from *pur* mutations [46, 47], lead to increased endogenous reactive oxygen species (ROS) generation [49, 50]. Additionally, PurF* exhibited a broader range of enriched GO terms among downregulated genes, including those related to the synthesis of various cellular components, compared to p*-βL. However, both mutants shared enrichment in terms related to various transport activities (Fig. 2D). The significant enrichment of GO terms related to the synthesis of various cellular components among the downregulated genes in PurF* may explain its slower growth compared to the WT and p*-βL (Fig. 2C). Overall, both mutants displayed broad physiological alterations, with PurF* exhibiting more severe changes (Fig. 2D). At the individual gene level, consistent with the qPCR results (Fig. 2A), p*-βL showed significantly increased expression of the TEM-type β-lactamase gene compared to the WT (Fig. 2D). Similarly, PurF*, which carries the Tyr610Frameshift mutation, demonstrated reduced expression of *purF*. Interestingly, *purF* expression was also reduced in p*-βL, though to a lesser extent (Fig. 2E). A notable change in both mutants, particularly in PurF*, was the significant upregulation of a gene encoding one of the entericidin components (Fig. 2E). These small, surface‐exposed lipoproteins, originally described as the entericidin toxin‐antitoxin pair [51, 52], have been shown to modulate cell aggregation, biofilm formation, motility, outer membrane vesicle release, and resistance to ROSs in *Xanthomonas citri* and *Agrobacterium tumefaciens* [53–55]. Investigating whether the overexpression of the entericidin gene contributes to the altered phenotype of the mutants, particularly PurF*, would be of interest. Additionally, it is notable that none of the mutants showed significant downregulation in the expression of surface or secreted proteins previously identified as beneficial to the host, including Amuc_1100 [42], the P9 protein [56], and Amuc_1409 [57] (Fig. 2E).

### *A. muciniphila* variants exhibited reduced capacity to mitigate host obesity

**Fig. 3:**
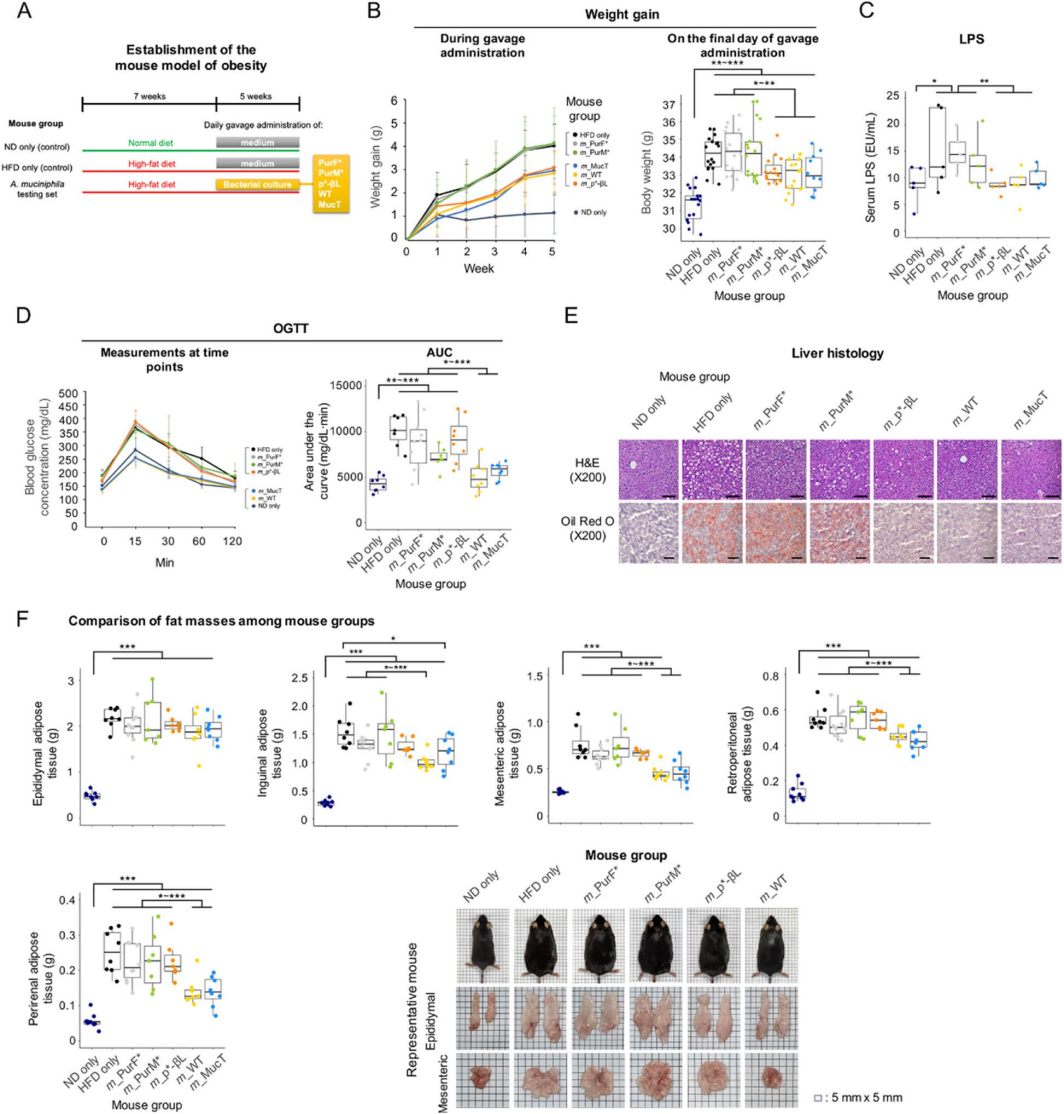
Antibiotics-selected *A. muciniphila* variants exhibited significantly reduced capacity to mitigate host obesity in the mouse model. **A** Establishment of the mouse model of obesity. Young (4-week-old) mice were fed either a normal (ND) or a high-fat diet (HFD) for 12 weeks. To test the effects of *A. muciniphila* strains, mice were subjected to gavage administration of bacterial cultures for the last 5 weeks. **B** Differential weight gain among the groups of mice during gavage administration. Mice administrated with PurF* (referred to as *m*_PurF*), PurM* (*m*-PurM*), the WT (*m*_WT), MucT (*m*_MucT), and p*-βL (*m*_p*-βL) are subjected to comparison. **C** Serum levels of LPS in mice. **D** Oral glucose tolerance test (OGTT) assays. Measurements at various time points and AOC (area of the curve) are compared among mouse groups. **E** Liver histology of mice. A representative sample was chosen from each group to illustrate distinct fat deposits. **F** Comparison of fat masses among mouse groups. Comparisons of epididymal, inguinal, mesenteric, retroperitoneal, and perirenal adipose tissues are shown. Pictures of representative mice from each group and their epididymal and mesenteric adipose tissues are shown. The whole set of the pictures is shown in Fig. S3. * *p* < 0.05, ** *p* < 0.01, *** *p* < 0.001 (Kruskal–Wallis test)

While increased expression of the TEM-type β-lactamase gene may primarily confer antibiotic resistance without significantly affecting bacterial physiology, the presence of additional downstream genes within the same operon (Fig. S2), such as the one encoding acyl-ACP-UDP-N-acetylglucosamine O-acetyltransferase, which may play a role in mucin degradation (Fig. 2C), suggests potential broader implications. Furthermore, mutations in *pur* genes are anticipated to have a pleiotropic effect due to a decrease in nucleotide levels, impacting various interconnected metabolic pathways and regulatory systems [46, 47]. To assess the potential impact of these antibiotics-selected mutations, we compared the host-beneficial capacity of the *A. muciniphila* variants with that of the wild-type strain in mice. To this end, we developed a mouse model of obesity by subjecting mice to a high-fat diet for 12 weeks (Fig. 3A). During the last 5 weeks of this period, these mice were subjected to daily gavage administration of either a bacterial culture or growth medium as a control (Fig. 3A). The tested strains included the type strain MucT (ATCC BAA-835), whose host-beneficial activity has been demonstrated [19, 21], the WT strain (the parental strain), and the variants: p*-βL (with the promoter mutation), PurF* (carrying *purF* (Tyr610Frameshift)), and PurM* (carrying *purM* (Gln9Pro)) (Fig. 3A). A group of mice fed a high-fat diet (HFD) without bacterial gavage administration (referred to as “HFD only” mice) exhibited a significantly higher increase in body weight compared to a group of mice fed a normal diet (ND) (referred to as “ND only” mice), highlighting the obesogenic effect of the high-fat diet (Fig. 3B). However, even among the groups of mice fed a high-fat diet, those gavaged with the WT strain or MucT (referred to as *m*_WT mice or *m*_MucT) exhibited significantly lower weight gain, consistent with the findings of previous studies [21] (Fig. 3B). Mice given strains did not show significant differences in appetite or food intake, suggesting that the observed differences in weight gain were not due to variations in the production of appetite-controlling hormones. Intriguingly, mice fed a high-fat-diet and gavaged with mutant strains PurF* (referred to as *m*_PurF*) or PurM* (referred to as *m*_PurM*), but not p*-βL (referred to as *m*_p*-βL), failed to exhibit the weight reduction exhibited by *m*_MucT or the *m*_WT mice (Figs. 3B). This underscores the reduced ability of the variants with *pur* mutations to mitigate host obesity. Furthermore, the weight gain in the mice strongly correlated with elevated serum lipopolysaccharide (LPS) levels (Fig. 3C), suggesting the onset of endotoxemia in these obese mice. Serum levels of other inflammation- or metabolic syndrome-associated factors, such as calprotectin, c-reactive protein (CRP), aspartate transferase (AST), alanine aminotransferase (ALT), triglycerides (TG), high-density lipoprotein cholesterol (HDL-C), and low-density lipoprotein cholesterol (LDL-C), were not significantly increased in mice fed high-fat diet compared to those fed a normal diet (data not shown). This suggests that our mouse model of obesity did not develop more serious conditions beyond obesity. In oral glucose tolerance tests (OGTTs) [44], *m*_PurF* and *m*_PurM* mice exhibited significantly compromised glucose tolerance compared to the *m*_ MucT and *m*_WT mice (Fig. 3D). Surprisingly, *m*_p*-βL mice also showed compromised glucose tolerance that was similar to *m*_PurF* and *m*_PurM* mice (Fig. 3D). The fat deposition displayed in the liver histology of obese mice, HFD only,* m*_PurF*, and *m*_PurM*, resembled that observed in hepatocellular steatosis, a hallmark of nonalcoholic fatty liver disease (NAFLD) [58, 59] (Fig. 3E). Fat deposition throughout the body was also evident in obese mice (Figs. 3F and S3). Specifically, these mice displayed significantly larger adipose tissues throughout the body than *m*_WT or *m*_MucT mice, with the exception that the epididymal adipose tissues were highly similar among all high-fat-diet-fed mice (Figs. 3F and S3). Remarkably, *m*_p*-βL mice also displayed distinct characteristics compared to *m*_WT or *m*_MucT mice, showing similar patterns to *m*_PurF* and *m*_PurM* mice (Fig. 3F), despite the absence of significant overall weight gain (Fig. 3B). Together, these findings indicate that all antibiotics-selected variants of *A. muciniphila* exhibited a reduced host-beneficial capacity, with purine metabolism defective mutants, PurF* and PurM*, showing an enhanced reduction than the promoter mutant p*-βL.

### Antibiotics-selected *A. muciniphila* variants may be globally prevalent in the human microbiome

**Fig. 4:**
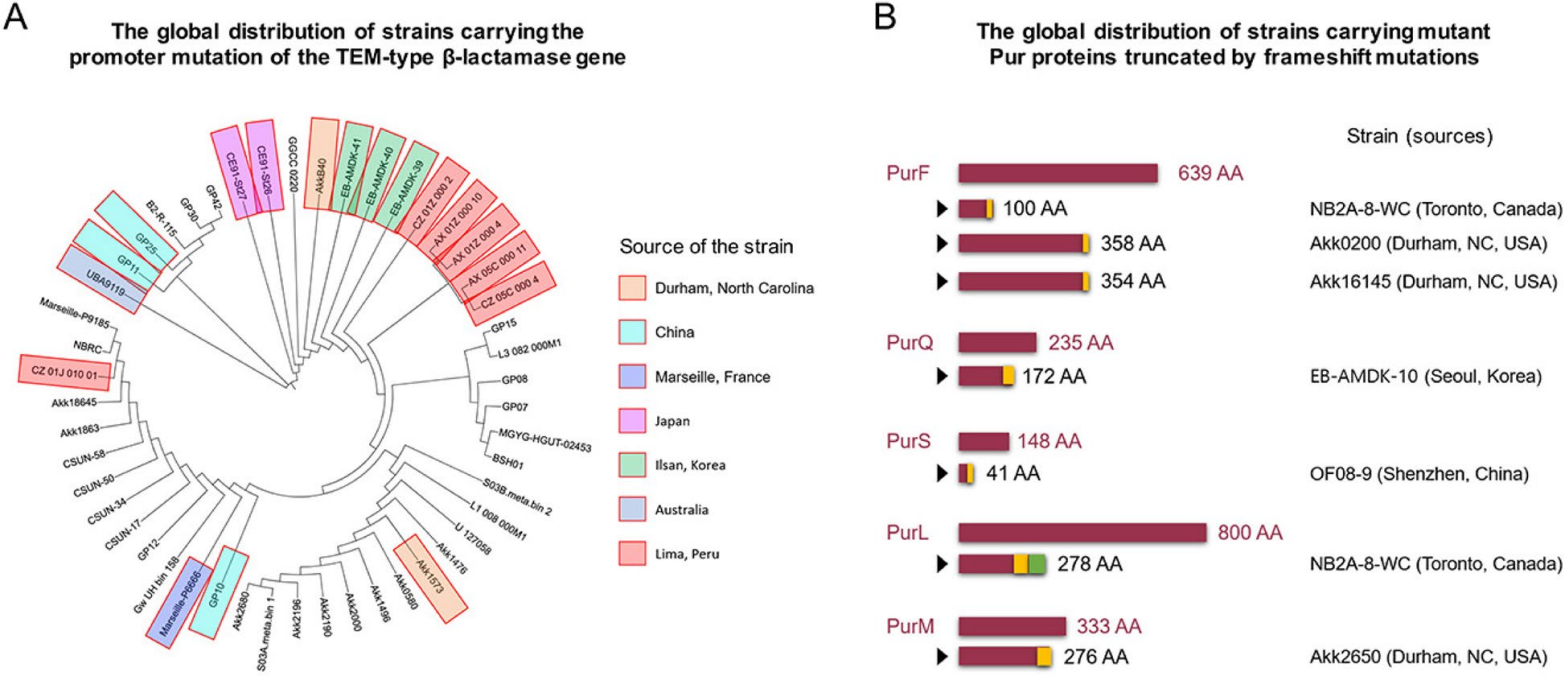
The global prevalence of the *A. muciniphila* variants in the human microbiome. **A** The global distribution of strains carrying the promoter mutation of the TEM-type β-lactamase gene. Strains carrying the A allele, corresponding to the mutation, at the SNP position in the −10 sequence of the promoter are denoted with colored boxes in the phylogenetic tree, which was constructed using TEM-type β-lactamase homologs. The sources of these strains are indicated. **B** The global distribution of *A. muciniphila* strains carrying mutant Pur proteins truncated by frameshift mutations. Sequences of *purF*, *purQ*, *purS*, *purL*, and *purM* from *A. muciniphila* strains were acquired from the NCBI database and subjected to analysis to identify mutant proteins. Yellow or green boxes displayed at the ends of the proteins represent amino acid sequences distinct from the wild-type sequences owing to frameshift mutations. The size of the proteins, strain names, and the sources of the strains are provided

To assess the worldwide prevalence of the SNP (single nucleotide polymorphism) resulting from the G to A substitution in the promoter of the TEM-type β-lactamase gene in *A. muciniphila* strains (Fig. 1A), we searched for the corresponding sequence in the National Center for Biotechnology Information (NCBI) database. As expected, there were only two possible nucleotide variations in this SNP: G and A. While the G allele, corresponding to the wild type, was more abundant, the A allele, corresponding to the mutation, was also prevalent globally among *A. muciniphila* strains across diverse regions, including the USA, France, Australia, Peru, Japan, China, and Korea [60, 61] (Figs. 4A and Table S1). Some closely related strains, as observed in the phylogenetic tree constructed using TEM-type β-lactamase homologs, shared this A allele, indicating that the mutation was inherited within these lineages (Fig. 4A). Conversely, some strains did not display the A allele shared by related strains (Fig. 4A), implying that their mutations have recently emerged. These discoveries suggest that the antibiotic selection observed in vitro has global in vivo prevalence and that the mutations have been inherited within *A. muciniphila* lineages in the human gut.

Subsequently, we identified potential signatures of antibiotics-selected *pur* gene mutations from the DNA sequences of *A. muciniphila* strains available in the NCBI database. While identifying that the promoter mutation was straightforward because of its specific base change at the designated location (a G-to-A substitution in the −10 sequence) (Fig. 1A), pinpointing mutations in the *pur* gene was more intricate due to their diverse nature. This diversity spans multiple target genes, such as *purF*, *purQ*, *purM*, and potentially other *pur* genes, and encompasses various modes of disruption, including missense, nonsense, and frameshift mutations (Figs. 1B and C). Therefore, distinguishing antibiotics-selected mutations from natural variations based solely on nucleotide sequences is challenging. Therefore, we focused on identifying frameshift and nonsense mutations in PurF, PurQSL, and PurM, which resulted in evident disruptions of the enzymes. Consequently, we discovered three strains carrying the truncated *purF* gene, two of which were closely related (Fig. 4B). Specifically, strain Akk16145 shared the same frameshift mutation found in *purF* as strain Akk0200, leading to the insertion of a stretch of different amino acids in PurF, starting at Gln349 (Fig. S4). Strain Akk16145 carried its own nonsense mutation downstream, resulting in the truncation from Gly355 (Fig. S4). This implies that strain Akk16145 is a descendant of strain Akk0200. Supporting this hypothesis, both strains originated from the same geographical location in Durham, NC, USA (Fig. 4B and Table S2). We identified a strain (NB2A-8-WC) carrying a mutant *purL* in which three consecutive mutations were observed: two frameshift mutations and a nonsense mutation (Figs. 4B and S4). It is conceivable that these mutations arose consecutively in this gene over generations, similar to the case of *purF* in strain Akk16145. The identification of numerous mutant *pur* genes from such limited database resources, even though we examined only a few *pur* genes and did not consider potentially abundant missense mutations, suggests that *A. muciniphila* strains carrying mutant *pur* genes are likely to be globally prevalent.

## Discussion

In this study, we investigated the potential selection of *Akkermansia* variants in the gut following exposure to penicillin and their effects on diet-induced obesity in mouse models. Two types of variants were identified: one with a mutation in the promoter of the TEM-type β-lactamase gene operon and the another with mutations in the *pur* genes, which encode the purine biosynthesis pathway. Both types of mutations significantly impacted the host-beneficial capacity of *A. muciniphila*, rendering it unable to protect against diet-induced obesity. This suggests a previously unknown mechanism by which antibiotics indirectly harm host health by disrupting key gut microbes like *A. muciniphila*.

We then assessed the global distribution of these mutations in human populations using publicly available *A. muciniphila* sequence data from the NCBI database. DNA sequences corresponding to both types of mutations were found to be widespread globally. However, further investigations were limited by a lack of detailed information about the source individuals, including factors such as antibiotic exposure, BMI, existing diseases, age, or gender. Despite this, the potential significance of these mutations in *A. muciniphila* within human populations warrants future studies to explore the extent to which these mutations are selected and inherited within lineages.

Four distinct phylogroups (AmI to IV) have been identified in *A. muciniphila* [62, 63]. According to this classification, the WT strain used in this study belongs to AMII, characterized by the presence of a corrin ring biosynthesis gene cluster [62], while the type strain MucT is classified under AMI [61]. The genome of the WT strain HM1 (GenBank CP169646, 3.2 Mb with approximately 2730 genes) is slightly larger than that of the MucT strain (GenBank NC_010655, 2.7 Mb with around 2240 genes). These strains share more than 1800 genes, including the TEM-type β-lactamase gene operon (Fig. S2) and the *pur* genes (Fig. 1B). The majority of the *A. muciniphila* population in the human gut belongs to these two phylogroups [61, 63]. Notably, strains GP20 [63] (GenBank GCA_002884975.1) and Akk2750 [61] (GenBank CP072018.1) belonging to AmIII and AmIV, respectively, also contain the TEM-type β-lactamase gene operon and *pur* genes. Despite substantial differences among the four phylogroups in traits such as growth rates on mucin, resistance to ambient oxygen, self-aggregation, and adhesion to epithelial surfaces [61], the similar effects of the mutations observed in this study are likely to be present across most *A. muciniphila* strains in the human microbiota, regardless of their phylogenetic lineage.

This novel mechanism, by which antibiotics affect host health by selecting *A. muciniphila* variants with reduced host-beneficial capabilities, has significant implications for understanding and developing new approaches to diagnosing and treating obesity and related metabolic conditions. It would be intriguing to explore whether this mechanism contributes to the increased incidence of early-onset obesity in infants and children following maternal antibiotic exposure before or during pregnancy, or their own exposure during early childhood [64, 65]. To extend these findings beyond humans, it is essential to investigate whether this mechanism also contributes to the unresolved observations in farm animals and mice, where exposure to sub-therapeutic doses of antibiotics has been linked to increased body weight and adipose mass [66–68]. Since the 1950s, antibiotics have been widely used in agriculture, and until recently, as growth promoters for farm animals [67, 68].

While the increased expression of TEM-type β-lactamase clearly accounts for reduced susceptibility to several β-lactam antibiotics (Fig. 2B), the mechanisms underlying disruptions in the *pur* genes are less well understood. Similar observations have been made, as disruptions in purine metabolism in bacterial pathogens have been linked to reduced antibiotic susceptibility and treatment failure in clinical settings [69–71]. Conversely, stimulating purine biosynthesis by limiting purines or increasing pyrimidines in the culture medium has been shown to induce cell death by raising ATP demand and accelerating metabolic rates [70, 72]. Additionally, PurF, which was mutated in the variant strains in this study, is a known target of the stringent response alarmone (p)ppGpp, which inhibits PurF and blocks purine synthesis in *E. coli* [73]. The stringent response is a critical mechanism for antibiotic tolerance in various bacteria [74–76]. Previous studies, including ours, have suggested that the stringent response is critical for gut bacterial survival in the gut environment [77] and may contribute to the long-lasting damage caused by antibiotics [18]. The mechanism underlying the reduced host-beneficial capacity remains unclear for both types of mutations. For the promoter mutation that induces genes in the TEM-type β-lactamase operon, it would be interesting to investigate whether the downstream genes, including the one encoding Acyl-ACP UDP N-acetylglucosamine O-acetyltransferase, contribute to the increased mucin layer degradation, as suggested by the growth experiment with N-acetyl-D-glucosamine supplementation. Notably, mucin degradation by *A. muciniphila* has been shown to regulate host lipid homeostasis by reducing the transcription of genes involved in cholesterol biosynthesis in the murine gut [78]. Regarding *pur* gene mutations, significant physiological changes are expected, yet the expression levels of bioactive molecules known for their beneficial effects on host health, such as the surface protein Amuc_1100 [36], the secreted protein P9, which stimulates glucagon-like peptide-1 (GLP-1) secretion [43], and Amuc_1409 [57], were not significantly reduced in the PurF* mutant. These findings suggest that other, as yet unidentified, mechanisms may be disrupted in the *pur* mutant*.*

While the promoter and *pur* gene mutations confer reduced susceptibility to antibiotics, they may not benefit bacteria in the absence of antibiotics, as they hinder in vitro growth, as demonstrated in this study. However, it remains unclear whether the slow in vitro growth observed in the mutants is also evident in the gut, where bacterial growth is inherently slow due to factors such as limited nutrient availability, heightened competition, the presence of bile acids, spatial constraints within niches, and host immune responses [79, 80]. Notably, qPCR analysis of fecal samples collected on the final day of bacterial administration revealed no statistically significant differences in the levels of either the promoter or *pur* gene mutants compared to the WT, indicating no evidence of impaired growth or colonization in the gut environment (Fig. S5). However, fecal bacterial levels may not fully reflect bacterial colonization or activity within the gut [81]. Even if the mutants were slightly less competitive in the gut, this alone is unlikely to account for their dramatic loss of ability to confer host-beneficial effects, as pasteurized *Akkermansia* cells have been shown to retain full efficacy [27, 42].

Importantly, the mutations seem to have been inherited within *A. muciniphila* lineages. This aligns with the understanding that mutations typically persist within the bacterial community owing to the slow reversibility of mutations at the community level [82]. This phenomenon is attributed to compensatory evolution, cost-free mutations, and genetic co-selection, even if the mutants themselves incur a fitness cost in the form of reduced growth rate [82]. Consequently, it is plausible that antibiotics-selected variants of *A. muciniphila* persist in human microbiota, potentially influencing human health. This further implies the possibility that these *A. muciniphila* variants have contributed to global epidemics of chronic conditions, offering insights into the missing links with altered microbiota in the modern human population.

## Conclusions

In recent decades, there has been a global surge in chronic conditions, and an altered gut microbiota in modern human populations has been suggested as a major contributing factor [1–5]. Antibiotic exposure has been proposed as a primary contributor to the cumulative alteration in the microbiota over generations [1, 3, 4, 13]. However, the mechanistic link between antibiotics-altered microbiota and chronic conditions has not been elucidated. In this study, we discovered that variants of the key gut microbe *A. muciniphila* can be selected and potentially replace its population upon antibiotic exposure. Importantly, these variants exhibit compromised ability to mitigate host obesity, and they are prevalent in the human microbiome worldwide. These results have enormous significance because they report a previously unexplored mechanism by which antibiotics influence human health by selecting variants of a key gut microbe, inevitably resulting in the loss of some host-beneficial capacities. Furthermore, this discovery may offer insights into the missing links between altered microbiota in modern humans and recent global epidemics of chronic conditions.

## Supplementary Information

Supplementary Material 1.

Supplementary Material 2.

Supplementary Material 3.

Supplementary Material 4.

Supplementary Material 5.

Supplementary Material 6.

Supplementary Material 7.

## Data Availability

No datasets were generated or analysed during the current study.
