# Supplementary figures and images for: Antibiotic-associated changes in Akkermansia muciniphila alter its effects on host metabolic health
Source: Microbiome. 2025 Feb 7;13:48. doi: 10.1186/s40168-024-02023-4 (PMC11804010; doi:10.1186/s40168-024-02023-4)

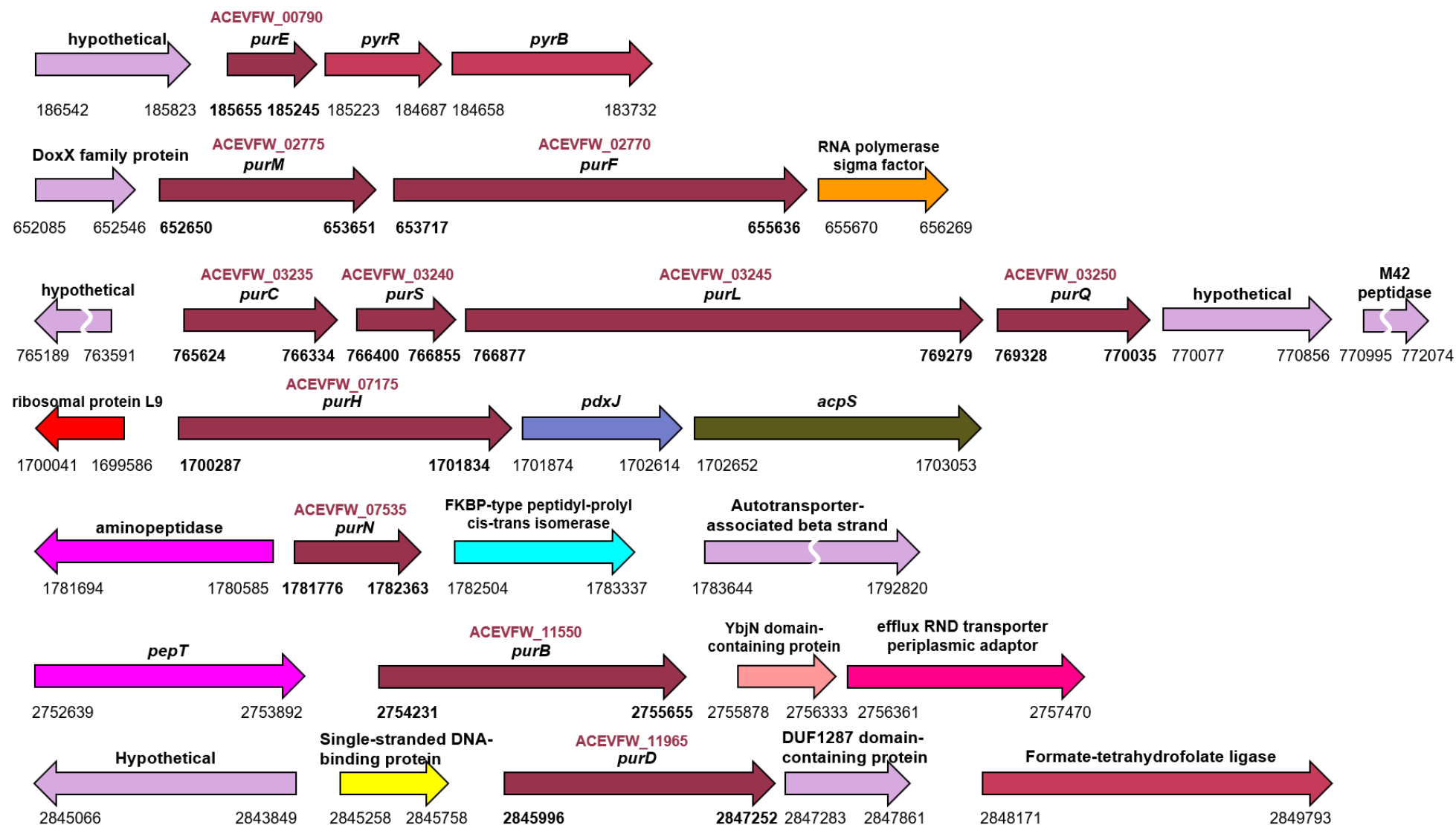

**Figure S1.** The organization of the *pur* genes in *A. muciniphila*.

Supplement: Supplementary file 2 — Supplementary Material 1. [file 40168_2024_2023_MOESM1_ESM.pdf]

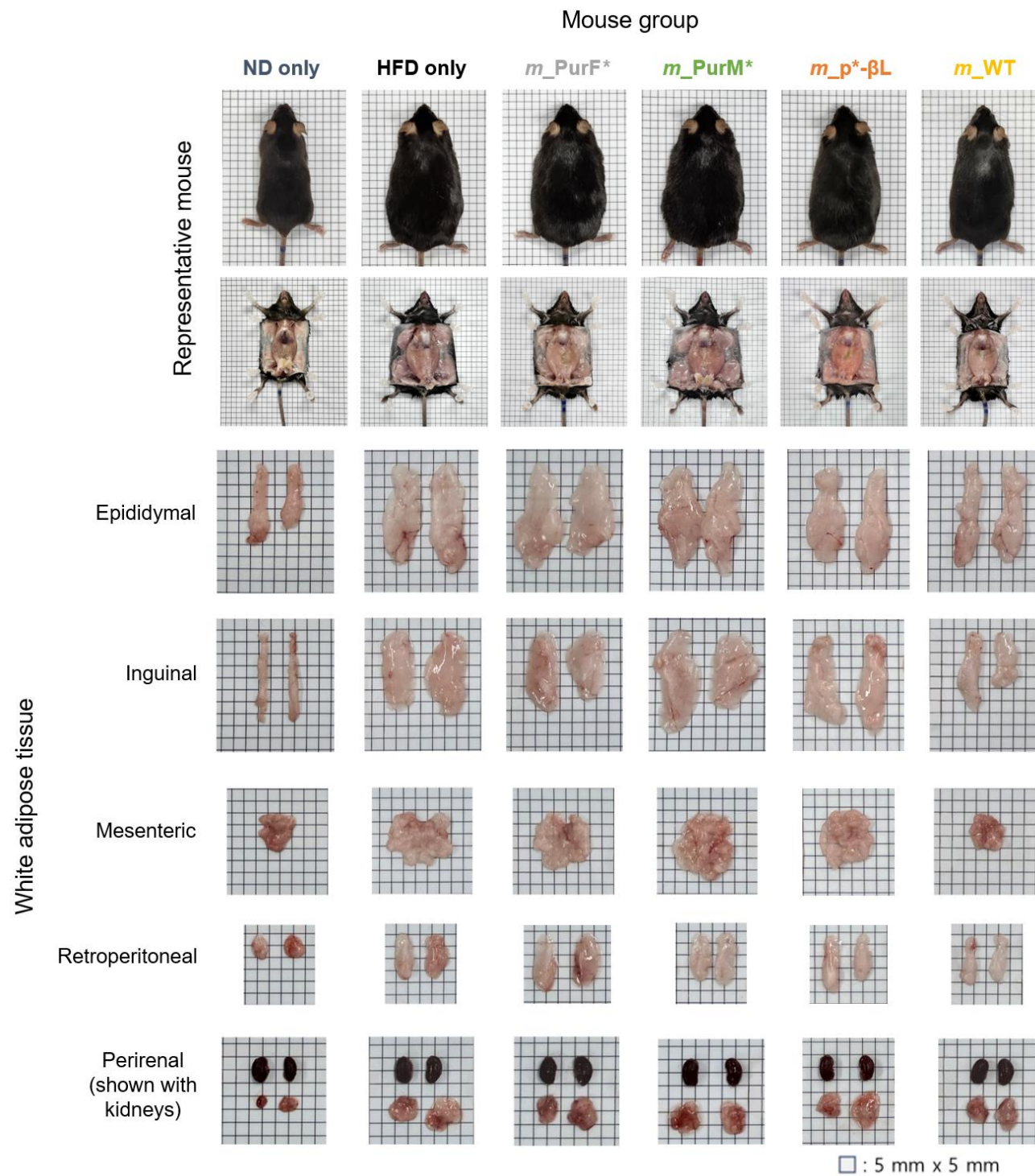

**Figure S3.** Pictures of mice and their adipose tissues representing each mouse group.

Supplement: Supplementary file 4 — Supplementary Material 3. [file 40168_2024_2023_MOESM3_ESM.pdf]
