# Supplementary material for: Antibiotic-associated changes in Akkermansia muciniphila alter its effects on host metabolic health
Source: Microbiome. 2025 Feb 7;13:48. doi: 10.1186/s40168-024-02023-4 (PMC11804010; doi:10.1186/s40168-024-02023-4)
Supplement: Supplementary file 3 — Supplementary Material 2. [file 40168_2024_2023_MOESM2_ESM.pdf]

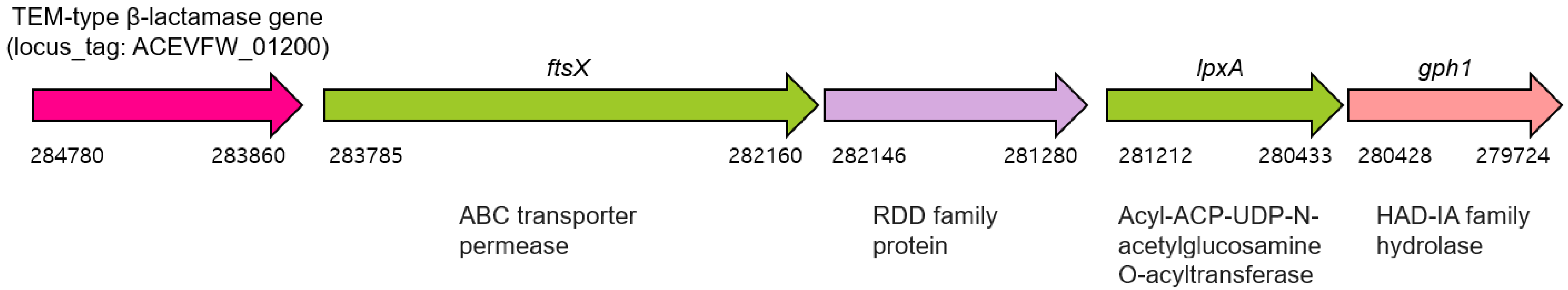

Figure S2. The organization of the operon containing the TEM-type β-lactamase gene (locus_tag: ACEVFW_01200). Five genes are in this operon.
