# Supplementary material for: Antibiotic-associated changes in Akkermansia muciniphila alter its effects on host metabolic health
Source: Microbiome. 2025 Feb 7;13:48. doi: 10.1186/s40168-024-02023-4 (PMC11804010; doi:10.1186/s40168-024-02023-4)
Supplement: Supplementary file 5 — Supplementary Material 4. [file 40168_2024_2023_MOESM4_ESM.pdf]

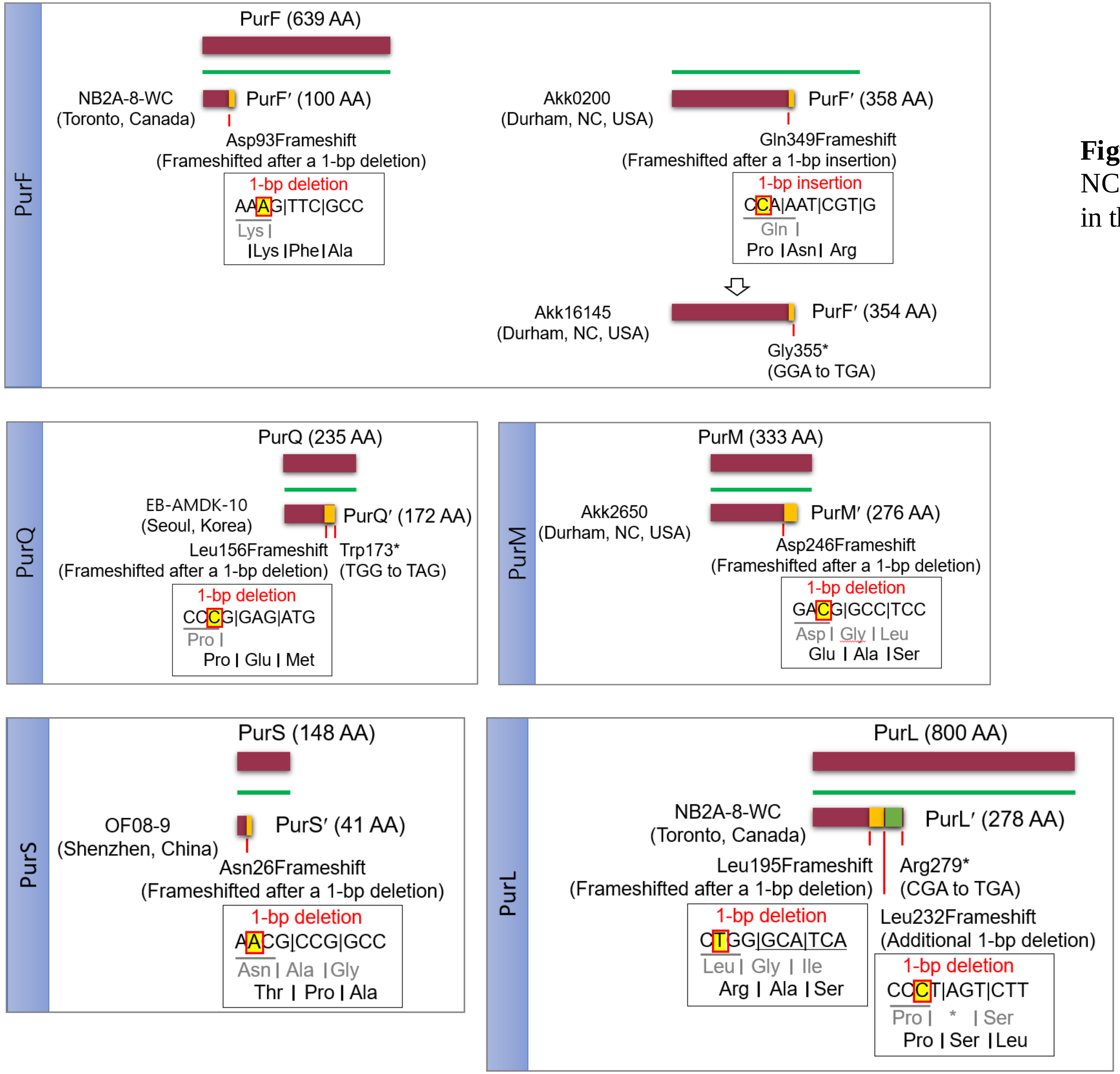

Figure S4. Details of mutations identified in *pur* genes from the NCBI database. Frameshift and nonsense mutations that resulted in the truncation of the encoded proteins are shown.
