# Supplementary material for: Antibiotic-associated changes in Akkermansia muciniphila alter its effects on host metabolic health
Source: Microbiome. 2025 Feb 7;13:48. doi: 10.1186/s40168-024-02023-4 (PMC11804010; doi:10.1186/s40168-024-02023-4)
Supplement: Supplementary file 6 — Supplementary Material 5. [file 40168_2024_2023_MOESM5_ESM.pdf]

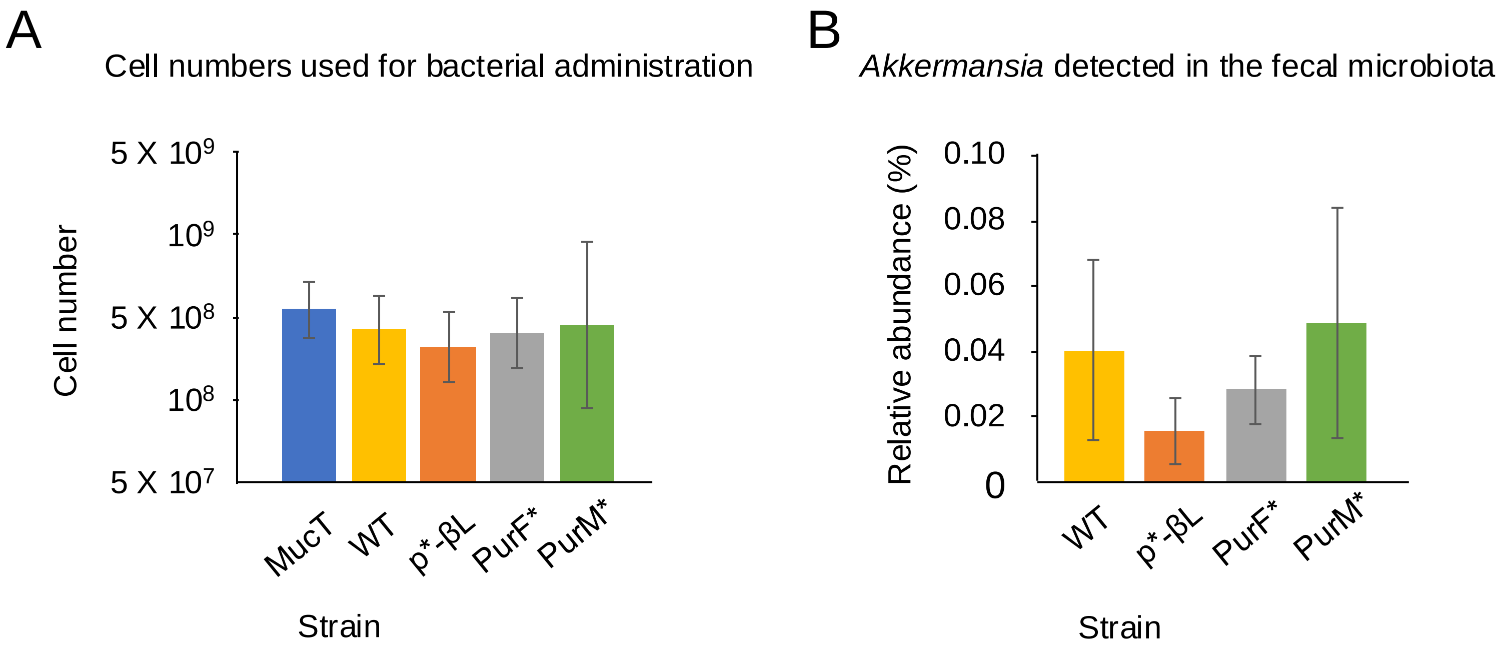

Figure S5. *Akkermansia* strains used in this study. **A.** Cell numbers used for bacterial administration. **B.** Quantitative PCR (qPCR) assays conducted with the fecal samples of mice collected on the final day of bacterial administration.
