# Supplementary material for: Antibiotic-associated changes in Akkermansia muciniphila alter its effects on host metabolic health
Source: Microbiome. 2025 Feb 7;13:48. doi: 10.1186/s40168-024-02023-4 (PMC11804010; doi:10.1186/s40168-024-02023-4)
Supplement: Supplementary file 7 — Supplementary Material 6. [file 40168_2024_2023_MOESM6_ESM.pdf]

Table S1. The strains carrying the TEM-type β-lactamase gene with or without the promoter mutation.

Strain (sample name)	BioSample	BioProject	Geographical location	Isolation source	w/ promoter mutation
Akk18645	SAMN18350259	PRJNA715455	USA:Durham, North Carolina	Human feces	
Akk0580	SAMN18350240	PRJNA715455	USA:Durham, North Carolina	Human feces	
Akk1476	SAMN18350247	PRJNA715455	USA:Durham, North Carolina	Human feces	
Akk1496	SAMN18350248	PRJNA715455	USA:Durham, North Carolina	Human feces	
Akk1573	SAMN18350249	PRJNA715455	USA:Durham, North Carolina	Human feces	Y
Akk1863	SAMN18350250	PRJNA715455	USA:Durham, North Carolina	Human feces	
Akk2000	SAMN18350261	PRJNA715455	USA:Durham, North Carolina	Human feces	
Akk2190	SAMN18350264	PRJNA715455	USA:Durham, North Carolina	Human feces	
Akk2196	SAMN18350265	PRJNA715455	USA:Durham, North Carolina	Human feces	
Akk2680	SAMN18350267	PRJNA715455	USA:Durham, North Carolina	Human feces	
AkkB40	SAMN18350269	PRJNA715455	USA:Durham, North Carolina	Human feces	Y
AX_01Z_000_10	SAMN04262587	PRJNA300541	Peru, Lima	Wastewater metagenome	Y
AX_01Z_000_4	SAMN04262587	PRJNA300541	Peru, Lima	Wastewater metagenome	Y
AX_05C_000_11	SAMN04261363	PRJNA300541	Peru, Lima	Wastewater metagenome	Y
B2-R-115	SAMN28745500	PRJNA843538	South Korea: Daegu	Human feces	
BSH01 scaf-11	SAMN08162545	PRJNA331216	China	Human gut	
CE91-St26 sequence1	SAMD00389904	PRJDB11902	Japan	Human feces	Y
CE91-St27 sequence1	SAMD00389905	PRJDB11902	Japan	Human feces	Y
CSUN-17 AmII_18	SAMN14614185	PRJNA609771	California, USA	Human feces (hispanic, age 32, M)	
CSUN-34 AmII_35	SAMN14614188	PRJNA609771	California, USA	Human feces (hispanic, age 22, M)	
CSUN-50 AmII_9	SAMN14614190	PRJNA609771	California, USA	Human feces (hispanic, age 23, F)	
CSUN-58 AmII_30	SAMN14614192	PRJNA609771	California, USA	Human feces (hispanic, age 33, F)	
CZ_01J_010_01	SAMN04261343	PRJNA300541	Peru, Lima	Wastewater metagenome	Y
CZ_01Z_000_2	SAMN04262587	PRJNA300541	Peru, Lima	Wastewater metagenome	Y
CZ_05C_000_4	SAMN04261343	PRJNA300541	Peru, Lima	Wastewater metagenome	Y
EB-AMDK-39	SAMN09287785	PRJNA473886	South Korea: Ilsan	Human feces	Y
EB-AMDK-40	SAMN09287787	PRJNA473887	South Korea: Ilsan	Human feces	Y
EB-AMDK-41	SAMN09288164	PRJNA473888	South Korea: Ilsan	Human feces	Y
GGCC_0220 NODE_22_length_27797_cov_491.049960	SAMN14738317	PRJNA628672	USA: North Carolina	Human feces	
GP07 scaf-6	SAMN08162547	PRJNA331216	China	Human feces	
GP08 scaf-8	SAMN08162548	PRJNA331216	China	Human feces	
GP10 scaf-5	SAMN08162549	PRJNA331216	China	Human feces	Y
GP11 scaf-3	SAMN08162550	PRJNA331216	China	Human feces	Y
GP12 scaf-8	SAMN08162551	PRJNA331216	China	Human feces	
GP15 scaf-7	SAMN08162552	PRJNA331216	China	Human feces	
GP25 scaf-3	SAMN08162553	PRJNA331216	China	Human feces	Y
GP30 scaf-11	SAMN08162554	PRJNA331216	China	Human feces	
GP42 scaf-1	SAMN08162555	PRJNA331216	China	Human feces	
Gw_UH_bin_158 Gw_UH_1408_length_27905_cov_11.424077	SAMN18120824	PRJNA524094	Germany: Greifswald	Wastewater metagenome	
L1_008_000M1 (Mother of infant L1_008)	SAMN08481510	PRJNA433393	USA: Pittsburgh, Magee-Womens Hospital of UPMC	Human feces	
L1_008_000M1 (Mother of infant #8)	SAMN17763793	PRJNA698986	USA: Pittsburgh, Magee-Womens Hospital of UPMC	Human feces	
L3_082_000M1 (Mother of infant #82)	SAMN17764044	PRJNA698986	USA: Pittsburgh, Magee-Womens Hospital of UPMC	Human feces	
Marseille-P6666 NODE_1_length_1816207_cov_81.7225	SAMN26693037	PRJNA816719	Marseille, France	Human feces	Y
Marseille-P9185 NODE_19_length_36025_cov_24.913226	SAMN26693038	PRJNA816719	Marseille, France	Human feces	
MGYG-HGUT-02453	SAMEA5851958	PRJEB33885	China	Human gut	
NBRC 115031 sequence11	SAMD00557632	PRJDB14606	Japan	Human feces	
S03A.meta.bin_1 k141_9684	SAMN10316505	PRJNA492716	Australia: Brisbane	Human feces	
S03B.meta.bin_2 k141_405	SAMN10316517	PRJNA492716	Australia: Brisbane	Human feces	
U_127058	SAMN21364986	PRJNA749124	Australia: Sydney, NSW	Phascolarctos cinereus (Koala) feces	
UBA9119 contig_12301	SAMN08020171	PRJNA417962	Australia	Human gut	Y
