# Supplementary material for: Antibiotic-associated changes in Akkermansia muciniphila alter its effects on host metabolic health
Source: Microbiome. 2025 Feb 7;13:48. doi: 10.1186/s40168-024-02023-4 (PMC11804010; doi:10.1186/s40168-024-02023-4)
Supplement: Supplementary file 8 — Supplementary Material 7. [file 40168_2024_2023_MOESM7_ESM.pdf]

Table S2. The *A. muciniphila* strains carrying mutant Pur proteins truncated by frameshift mutations.

Strain (sample name)	ACCESSION	DBSOURCE	Gene	BioSample	BioProject	Geographical location	Source	w/ frameshift	URL
NB2A-8-WC	UQT45456.1	CP097283.1	*purF*	SAMN28102047	PRJNA835435	Toronto, Canada	Human feces	Y	https://www.ncbi.nlm.nih.gov/ipg/UQT45456.1
Akk0200	WP_257228631.1	NZ_CP072050.1	*purF*	SAMN18350234	PRJNA715455	Durham, North Carolina, USA	Human feces	Y	https://www.ncbi.nlm.nih.gov/ipg/WP_257228631.1
Akk16145	WP_257228177.1	NZ_CP072031.1	*purF*	SAMN18350254	PRJNA715455	Durham, North Carolina, USA	Human feces	Y	https://www.ncbi.nlm.nih.gov/ipg/WP_257228177.1
EB-AMDK-10	WP_260735522.1	NZ_CP025825.1	*purQ*	SAMN08329147	PRJNA429085	Seoul, Korea	Human feces	Y	https://www.ncbi.nlm.nih.gov/ipg/WP_260735522.1
OF08-9	WP_270589558.1	NZ_JAQEVJ010000016.1	*purS*	SAMN31809641	PRJNA903559	Shenzhen, China	Human feces	Y	https://www.ncbi.nlm.nih.gov/ipg/WP_270589558.1
NB2A-8-WC	UQT45595.1	CP097283.1	*purL*	SAMN28102047	PRJNA835435	Toronto, Canada	Human feces	Y	https://www.ncbi.nlm.nih.gov/ipg/UQT45595.1
Akk2650	WP_257227633.1	NZ_JAGFOC010000002.1	*purM*	SAMN18353368	PRJNA715455	Durham, North Carolina, USA	Human feces	Y	https://www.ncbi.nlm.nih.gov/ipg/WP_257227633.1
